# Generating drought- and salt-tolerant tomato germplasm through bioengineering of the ethylene response factor SlERF.J2

**DOI:** 10.48130/els-0026-0003

**Published:** 2026-06-27

**Authors:** Ya-Nan Chen, Si-Yu Huang, Zi-Yi Wang, Yi-Han Sun, Bao-Ying Wang, Jun Gao, Fa-Xiang Wan, Jian-Ping Tao, Jing Zhuang, Guang-Long Wang, Ai-Sheng Xiong

**Affiliations:** 1 Faculty of Life Science and Food Engineering, Huai'an University, Huai'an, Jiangsu 223003, China; 2 State Key Laboratory of Crop Genetics & Germplasm Enhancement and Utilization, College of Horticulture, Nanjing Agricultural University, Nanjing, Jiangsu 210095, China

**Keywords:** SlERF.J2, Bio-engineering, Drought stress, Salt stress, Tomato

## Abstract

Drought and salinity are key abiotic constraints limiting the growth, development, and yield of tomato (*Solanum lycopersicum*). Ethylene response factors are crucial regulators that govern plants' adaptation to diverse abiotic and biotic stress stimuli. Nevertheless, the exact role of *SlERF.J2* in abiotic stress resistance remains unclear. In this study, clustered regularly interspaced short palindromic repeats (CRISPR)/CRISPR-associated protein 9 gene-editing technology was used to generate *slerf.j2 *knockout tomato lines to clarify the biological function of *SlERF.J2* in regulating tomato's responses to drought and salt stress. The results revealed that deletion of *SlERF.J2* markedly enhanced tomato seedlings' resistance to polyethylene glycol 6000-induced drought, mannitol, and salt stresses. Compared with the wild-type (WT), the knockout lines exhibited significantly increased peroxidase activity and leaf relative water content, as well as markedly decreased contents of hydrogen peroxide and malondialdehyde. After stress treatment, the expression levels of genes associated with the stress response, flavonoid biosynthesis, chlorophyll biosynthesis, light response, cell division, and hormone biosynthesis in SlERF.J2-edited tomato lines were significantly higher than those in WT plants. A yeast one-hybrid assay confirmed the direct interaction between SlERF.J2 and the *SlCPS* promoter. Collectively, this study clarifies the important function of *SlERF.J2* in regulating drought and salt stress tolerance in tomato, providing genetic resources for tomato breeding.

## Introduction

Global agricultural productivity and food security are seriously jeopardized by abiotic adversities, especially drought and soil salinization^[1]^. As a globally important cash vegetable crop^[2]^, tomato (*Solanum lycopersicum* L.) is highly sensitive to water deficit and high-salt environments during its growth and development^[3−4]^. Abiotic stresses induce numerous detrimental physiological responses in tomato plants, including stomatal closure, suppressed photosynthetic capacity, excessive reactive oxygen species (ROS) accumulation, and the eventual deterioration of crop yield and quality^[5]^. Accordingly, characterization of the core regulatory genes underlying stress tolerance and dissection of their molecular mechanisms are essential for developing stress-resistant tomato cultivars via genetic manipulation. Existing studies have demonstrated that multiple transcription factor families, including ethylene response factors (ERFs), WRKY, NAC, and MYB, play vital roles in regulating plants' growth and development as well as abiotic stress responses^[6−7]^.

As an important subfamily of the AP2/ERF superfamily, ERF transcription factors are critical regulators of plants' defense responses to abiotic stresses^[8−9]^. ERF proteins harbor a highly conserved AP2/ERF functional domain^[10]^, which mediates their specific binding to *cis*-regulatory motifs in the promoters of downstream stress-responsive genes, such as *DRE* (*dehydration-responsive element*) and *ERE* (*ethylene-responsive element*). Through such binding characteristics, ERFs regulate plants' stress resistance by activating or repressing transcription of the target genes^[11]^.

Growing research has revealed that ERF family proteins function as core regulators in the signaling cascade governing plants' resistance to diverse abiotic stresses. In rice (*Oryza sativa*), OsDREB2B negatively regulates growth through *OsAP2-39*/*OsWRKY21*-mediated regulation of gibberellin (GA) metabolism, thereby reducing GA content and plant height^[12]^. *OsEREBP1* positively regulates the JA (jasmonic acid) and ABA (abscisic acid) signaling modules, which further strengthens rice's adaptability to diverse abiotic and biotic stressors^[13]^. Overexpression of *OsERF71* regulates ABA-responsive and proline biosynthesis genes, enhances ABA sensitivity, and promotes proline accumulation, thereby reducing water loss in rice and improving drought tolerance^[14]^. Overexpression of *OsDREB2B* significantly enhances drought tolerance in rice^[15]^. When subjected to low-temperature conditions, *OsERF096* exerts regulatory effects on numerous vital physiological routes, covering carbohydrate conversion between starch and sucrose and phytohormone signal cascades. This transcription factor alters the sugar content and auxin (IAA) accumulation in transgenic rice, regulates the transcriptional expression of *OsIAA* and *OsARF* genes, and thereby enhances rice's resistance to cold stress^[16]^. The *OsERF19* transcription factor enhances rice's tolerance to salt stress by activating the expression of *OsOTS1* and *OsNCED5*, thereby promoting ABA signal transduction pathways^[17]^.

In *Arabidopsis thaliana*, overexpression of *AtERF15* enhances plants' responsiveness to ABA, salt stress, and osmotic stress, improves drought tolerance, and activates ABA signaling pathways^[18]^. After being transferred into *A. thaliana*, *ZmEREB20* enhances plants' responsiveness to ABA by regulating the transcription of genes involved in the ABA–GA signaling pathway, and delays seed germination under salt stress conditions^[19]^. *AtERF71*/*HRE2* functions as a transcription factor that positively regulate *A. thaliana*’s responses to osmotic and hypoxic stresses^[20−21]^. Excessive expression of *ERF012* improves plants' tolerance of salt stress, drought stress, and heavy metal stress^[22]^. ABA can induce the degradation of *ERF.D2*, which, in turn, stimulates the transcription of core genes in the JA signaling pathway, further increasing the endogenous JA content in plants, and enhancing tomato's tolerance of drought stress^[23]^.

The tomato AP2/ERF family consists of 164 members. To date, several stress-associated ERF family genes have been identified in tomato, including *SlERF.A3*, *SlERF.B2*, *SlERF.C1*, *SlERF.E2*, *SlERF.E3*, *SlERF.F5*, and *SlERF.G2*^[9]^. The ERF.J subfamily in tomato consists of three members, namely *SlERF.J1*, *SlERF.J2*, and *SlERF.J3*. To date, no functional characterization has been reported for these three genes, leaving their biological roles largely unexplored. Uncovering their functions will fill the research gap in ERF-mediated stress-response regulatory networks and provide candidate genes for breeding stress-resistant tomato varieties. Sequence homology analysis revealed that *AtERF5* from *A. thaliana*, which shows high homology to *SlERF.J2*, has been verified to enhance salt tolerance in plants^[24]^. On the basis of an analysis of gene expression patterns, we inferred that *SlERF.J2* may participate in stress responses and hormone signaling pathways in tomato.

*SlERF.J2* is categorized as a member of the tomato AP2/ERF-type transcription factors, yet its exact biological roles in modulating the crop's adaption to abiotic challenges have not been fully elucidated. In this study, we isolated and cloned the *SlERF.J2* gene. Transcript abundance analysis suggests that this gene is likely involved in regulatory pathways to mediate tomatos acclimation to stresses. To further explore the biological function of *SlERF.J2*, we generated *SlERF.J2* knockout transgenic tomato plants. *SlERF.J2* knockout significantly improved tomato seedlings' performance under salt and mannitol stress. Meanwhile, under drought and salt conditions, this gene deletion enhanced plants' abiotic stress resilience through regulating physiological traits and the expression of stress genes. These results uncover the hitherto unclear functions of *SlERF.J2* in coping with abiotic adversities and enrich our existing understanding of AP2/ERF-dependent regulatory mechanisms underlying abiotic stress tolerance in plants. Furthermore, newly created stress-resistant tomato germplasm lays a genetic foundation for molecular improvement of stress-resistant tomato lines.

## Materials and methods

### Sequence analysis of *SlERF.J2*

Protein sequences encoded by *SlERF.J2* and its homologous counterparts from various plant species were retrieved from the National Center for Biotechnology Information (NCBI) database. The phylogenetic tree was constructed using the neighbor joining method in MEGA 6.0 software^[25]^. Multiple sequence alignment of ERF-family proteins was carried out using DNAMAN software.

The 3,000-bp upstream promoter region of *SlERF.J2* was extracted from the Sol Genomics Network database. The corresponding *cis*-regulatory elements were subsequently identified and characterized via the PlantCARE web server.

### Plant materials and growth conditions

The wild-type (WT) tomato cultivar Ailsa Craig (marked as AC^++^) served as the recipient material to develop transgenic lines. The plant materials used in this study were obtained from the laboratory of Prof. Guoping Chen, College of Bioengineering, Chongqing University. Tomato plants were cultivated in growth chambers under a temperature regime of 28 °C during the 16-h light period and 18 °C during the 8-h dark period. The culture environment had 70% relative humidity and a photosynthetic photon flux density of 250 μmol·m^−2^·s^−1^.

### Analysis of expression patterns

We analyzed the hormone-induced expression patterns of *SlERF.J2* by treating 5-week-old WT tomato plants with four exogenous phytohormones (50 μM methyl jasmonate [MeJA], 50 μM IAA, 50 μM ABA, and 100 μM GA₃). Each group contained three biological replicates. Leaf specimens were sampled across distinct time intervals within one day to facilitate further experimental determinations.

We investigated how *SlERF.J2* responds to multiple abiotic stresses at the transcription and physiology levels using 5-week-old WT tomato seedlings. Salt treatment was performed via soaking the roots in a 300 mM NaCl solution following a published protocol^[26]^. Drought stress was simulated by uprooting the seedlings, rinsing off residual soil from the root system, and then placing whole 8-week-old seedlings on dry filter paper. To impose thermal and chilling stresses separately, young tomato seedlings were exposed to 42 and 4 °C ambient conditions, respectively. Each treatment had three biological replicates to ensure the results' reliability. Leaf samples were gathered at the designated time points after treatment for subsequent analysis^[27]^. After packaging in aluminum foil, leaf specimens underwent ultra-rapid freezing in liquid nitrogen prior to long-term preservation at −80 °C, which keeps the bioactivity of nucleic acids and proteins intact for follow-up molecular characterization.

### Construction of the vector and plant transformation

Using the TargetDesign platform (https://skl.scau.edu.cn/targetdesign), we screened an editing target in Exon 1 of the *SlERF.J2* gene and then assembled the CRISPR/Cas9-*SlERF.J2* expression plasmid. The generated vector was delivered into *Agrobacterium tumefaciens* LB4404, which was then used to transform WT tomato plants through *Agrobacterium*-mediated infection^[28]^. Seedlings resistant to 50 mg·L^−1^ kanamycin were preliminarily selected, and subsequent NPTII-targeted (Neomycin phosphotransferase II) PCR (Polymerase chain reaction) was used to verify successful T-DNA integration. Subsequently, sequencing analysis of the *SlERF.J2* coding sequence was conducted to identify the exact knockout genotype.

### Total RNA extraction and quantitative reverse-transcription–PCR analysis

Frozen samples were used for total RNA extraction via the TRIzol method as described earlier^[29]^. A micro-spectrophotometer (CFX CONNECT, USA) was applied to detect the RNA concentration. The A_260_/A_280_ (Absorbance at 260 nm / Absorbance at 280 nm) ratios of all samples fell in the range of 1.8–2.0, indicating intact pure RNA. Total RNA was reverse-transcribed into cDNA following the instructions of commercial kits.

The quantitative reverse transcription–PCR (qRT-PCR) reactions were implemented on the CFX96 real-time thermal cycler (Bio-Rad, United States). The reaction system and procedures were performed according to previous studies^[30]^. The stably expressed *SlCAC* (*Solyc08g006960*) gene was selected as a reference gene^[31]^. Relative gene expression was calculated via the 2^−ΔΔCᴛ^ method^[32]^. Detailed primer information is shown in Supplementary Table S1.

### Seed germination assay under polyethylene glycol 6000 treatment

Seeds subjected to surface sterilization and vernalization were placed in Petri dishes lined with filter paper, followed by the addition of 5 mL of polyethylene glycol (PEG) 6000 solutions at concentrations of 0, 3%, 6%, and 9%, respectively. The mannitol concentrations were chosen according to previous studies. To sustain stable osmotic stress conditions, 1 mL of a corresponding concentration of a PEG6000 solution was supplemented every 48 h. Each genotype under the individual stress treatments was analyzed with three technical replicates. Daily germination statistics were recorded after sowing, ending after five successive days with no newly germinated seeds. The entire assay was conducted with three independent biological replicates. The recorded experimental data were used to calculate the following parameters^[33]^.

$ \mathrm{Seed\ germination\ rate\ ({\%})=\dfrac{Final\ no.\ of\ germinated\ seeds}{Total\ no.\ of\ sown\ seeds}\times100{\%}} $

$ \mathrm{Mean\ germination\ time\ (d)=\dfrac{\textstyle\sum (No.\ of\ seeds\ germinated\ on\ day\ n\times n)}{Final\ total\ no.\ of\ germinated\ seeds}} $

\begin{equation*}\begin{split}&\mathrm{Germination\ energy\ ({\%})}=\\&\rm\quad\dfrac{No.\ of\ seeds\ germinated\ at\ the\ peak\ germination\ stage}{Total\ no.\ of\ sown\ seeds}\times 100{\%}\end{split}\end{equation*}

### Mannitol stress treatment for tomato seedlings

Seeds were sterilized and vernalized, then cultured under dark shaking conditions to induce radicle germination. Uniformly germinated seeds were placed on Murashige and Skoog (MS) media supplemented with 0, 150, and 300 mM of mannitol and cultured in a light incubator. The mannitol concentrations were chosen according to previous studies. Obvious growth differences appeared between the WT and transgenic plants, and then the seedlings' root length was measured. Each treatment had three biological replicates to ensure the results' reliability.

### Salt stress treatment for the seed germination and seedling growth assay

After sterilization and vernalization as a pretreatment, seeds were cultured in a shaking incubator at 28  °C and 120 rpm in darkness until germination occurred. Seeds with a consistent germination status were transplanted to MS media with a concentration gradient of 0, 50, and 100 mM for cultivation under illumination. The selection of NaCl concentrations was according to previous studies. Growth data were recorded after phenotypic differences appeared, with three technical replicates for each genotype in all treatments.

### Salinity and drought stress tolerance experiment

Seedlings with identical germination performance were inoculated on MS substrates supplemented with graded NaCl concentrations for culturing under light. Samples were harvested after the salt treatment for subsequent experiments, with three biological replicates.

To assess drought tolerance, all seedlings were fully irrigated under identical conditions prior to the water deficit treatment^[34]^. Every experimental group was configured with triplicate biological samples to enhance the credibility of the measured results.

### Measurement of relative electrical conductivity, relative water content, malondialdehyde and hydrogen peroxide content, and peroxidase activity

To determine the relative electrical conductivity (REC), leaf discs were punched from homologous positions on the sampled leaves. Thirty round leaf slices were obtained with a leaf punch and submerged into test tubes with 10 mL of pure water. The first conductivity value (R_1_) was recorded after 12 h of static incubation. Thereafter, the tubes were subjected to boiling for 30 min, and R_2_ was tested once the specimens had returned to room temperature. REC was computed using the following formula: REC = (R_1_/R_2_) × 100%.

Leaf malondialdehyde (MDA) content was assayed via the thiobarbituric acid (TBA) method^[35,36]^. Samples were placed in 2-mL centrifuge tubes with grinding beads and 2 mL of 10% tricarboxylic acid (TCA), then fully ground. Samples were centrifuged at 15,000 × *g* for 5 min. An aliquot of 1 mL of the upper liquid was added to 4 mL of 0.5% TBA, heated at 95 °C for 30 min, and then chilled quickly. Spectrophotometric absorbance was quantified at 450, 532, and 600 nm.

The peroxide (H_2_O_2_) content in tomato leaves was quantified via the potassium iodide (KI) method. For sample preparation, a 0.1-g sample of fresh leaves was fully pulverized using 1.5 mL of a precooled 0.1% TCA buffer; the resulting homogenate was spun at 12,000 × *g* and 4 °C for 15 min. The collected supernatant was blended successively with 0.5 mL of a phosphate buffer solution and 1 mL of a working solution of KI. After steady incubation at 28 °C for 1 h, the absorbance of the reaction liquid was detected at 390 nm, and the actual H_2_O_2_ content was calculated according to an established standard curve^[37]^.

The determination of leaf relative water content (RWC) relied on three weight indices, namely fresh weight, water-saturated weight, and dehydrated dry weight. Peroxidase (POD) activity was assayed to previously reported methods with minor modifications^[38,39]^.

Each treatment had three biological replicates to ensure the results' reliability.

### Histochemical staining

Leaf samples were subjected to trypan blue and diaminobenzidine (DAB) staining with an optimized protocol adapted from previous reports^[25]^. Specimens were submerged in 0.1% trypan blue reagent, underwent water bath heating at 95 °C for 10 min and dark incubation lasting for half an hour; afterwards, 95% ethanol was applied to remove the pigment. Leaves were soaked in a 0.1% DAB solution and shaken at 50 rpm for 4 h at 28 °C, then immersed in 95% ethanol and boiled for 15 min to remove chlorophyll. Experiments were performed with three biological replicates.

### Yeast one-hybrid assays

The yeast one-hybrid (Y1H) assays were performed using the Matchmaker Gold Y1H System (TaKaRa). PCR cloning was used to obtain the full-length *SlERF.J2* open reading frame (ORF), which was subsequently recombined with pGADT7 as the prey construct. The *SlCPS* promoter sequence was subcloned into pAbAi to produce the corresponding bait plasmid. Linearized recombinant pAbAi-pro*SlCPS* was transformed into Y1H gold yeast cells. Screening of the lowest inhibitory ABA dosage was performed to rule out self-activation by the bait vector. Competent yeast was transformed with prey plasmids and spread onto two types of SD/-Leu (synthetic dropout medium without leucine) solid media (with and without ABA). pAbAi-p53 paired with pGADT7-p53 was set as a positive experimental control. Following incubation at 30 °C for 2 to 3 d, colony growth was observed. Detailed primer information can be found in Supplementary Table S2.

### Statistical analysis

All statistical computations were completed in SPSS version 26.0. Independent sample Student's *t*-test was used to compare differences between two experimental groups; statistical thresholds were set at *p* < 0.05 for significant variations and *p* < 0.01 for highly significant variations. For intergroup multiple comparisons, one-way analysis of variance (ANOVA) followed by Tukey's *post hoc* test was utilized to determine significant differences at the *p* < 0.05 level. Three independent biological replicates were arranged per experimental group to guarantee the credibility of the data obtained.

## Results

### Molecular characterization of SlERF.J2

**Figure 1:**
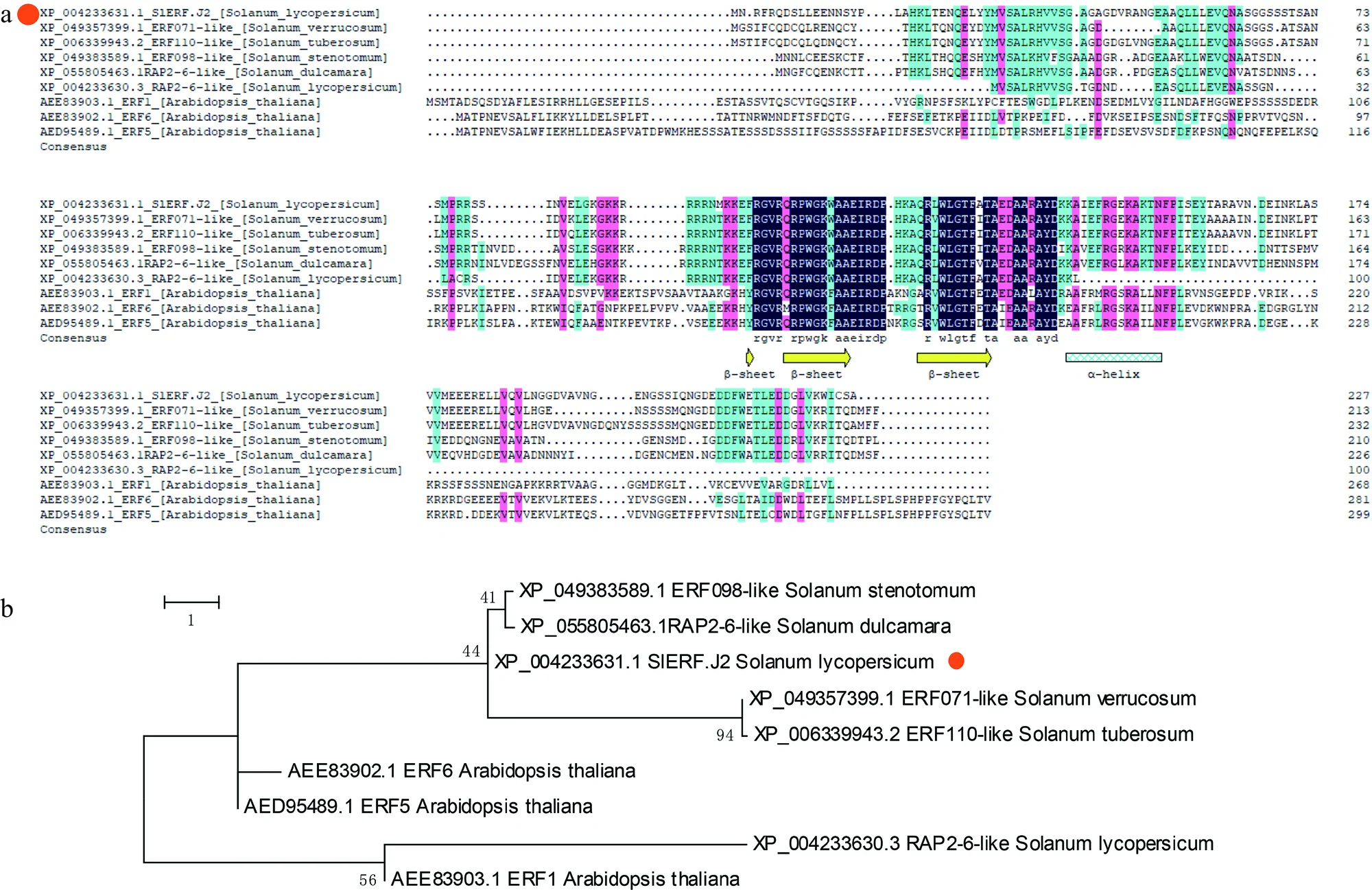
Sequence analysis of SlERF.J2. (a) SlERF.J2 and other ERF protein sequences were aligned comparatively. Conserved amino acid residues are marked with black shading. The protein structure of SlERF.J2 contained three *β*-sheets and one *α*-helical domain. (b) Phylogenetic reconstruction relied on the neighbor-joining approach with 1,000 bootstrap repetitions; *SlERF.J2* is labeled with a circle. The relevant protein accession numbers are SlERF.J2 (XP_004233631.1), SsERF098-like (XP_049383589.1), SdRAP-like (XP_055805463.1), SvERF071-like (XP_049357399.1), StERF110-like (XP_006339943.1), AtERF6 (AEE83902.1), AtERF5 (AED95489.1), SlRAP-6-like (XP_004233630.3), and AtERF1 (AEE83903.1).

For the characterization of the SlERF.J2 protein (XP_00423361.1) and its evolutionary associations, we conducted multiple sequence alignment and constructed a phylogenetic tree. Multiple sequence alignment showed that SlERF.J2 shares high sequence similarity with ERF/RAP2.6-like proteins from *Solanum* species (*S. stenotomum*, *S. tuberosum*, *S. lycopersicum*) and ERF proteins from *A. thaliana* (Fig. 1a). The AP2/ERF domain exhibited high conservation across all aligned sequences. The *β*-sheet and *α*-helix secondary structures, which form the core of the AP2/ERF DNA-binding motif, exhibited nearly identical amino acid composition in the *Solanum* orthologs, indicating functional conservation of this domain in the tomato ERF protein SlERF.J2.

Phylogenetic analysis was conducted to clarify the evolutionary links of SlERF.J2. The corresponding phylogenetic tree was generated using ERF homologs from *Solanum* crops and the full ERF protein dataset of *Arabidopsis* (Fig. 1b). The results showed that SlERF.J2 clustered tightly with other *Solanum* ERF/RAP2.6-like proteins, forming a monophyletic clade distinct from *Arabidopsis* ERFs (AtERF1, AtERF5, AtERF6). This clade included *S. stenotomum* ERF098-like, *S. tuberosum* ERF110-like, and *S. lycopersicum* RAP2-6-like proteins, demonstrating that SlERF.J2 is a *Solanum*-specific ERF ortholog and is evolutionarily distinct from *Arabidopsis* ERF subfamilies.

### Expression patterns of the *SlERF.J2* gene

**Figure 2:**
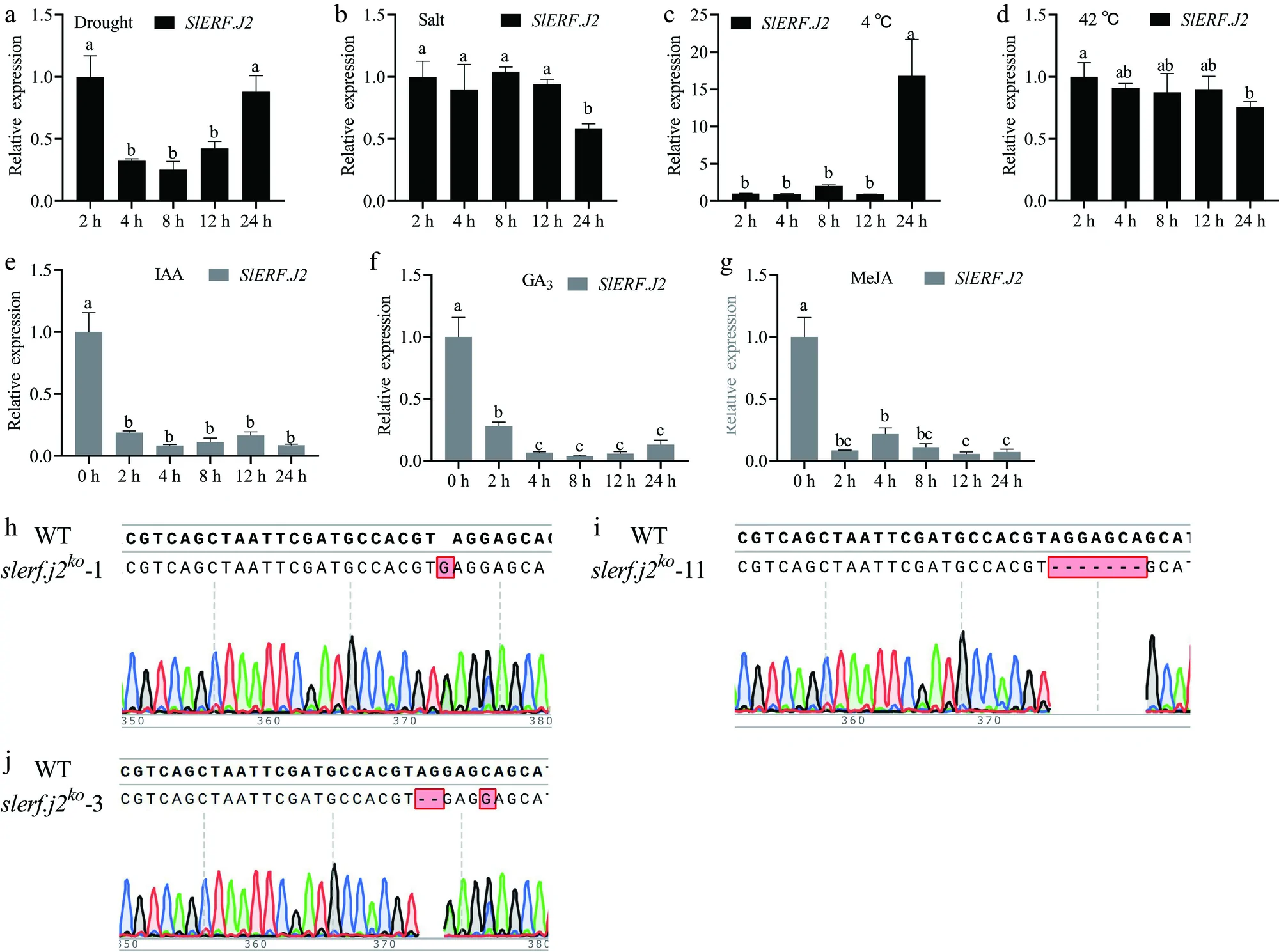
Transcript abundance of *SlERF.J2* under abiotic constraints and hormone stimuli, alongside functional verification for CRISPR-generated *slerf.j2* knockout materials. (a)–(g) Time-related expression patterns of *SlERF.J2* under different treatments: Drought, salt, 4 °C (cold), 42 °C (heat), IAA, GA_3_, and MeJA. Values are presented as the mean ± standard error (SE) from three independent biological replicates. Significant differences among groups were determined by one-way ANOVA and are marked with disparate lowercase letters. (h)–(j) Validation of the mutation sites in *slerf.j2* knockout lines (ko-1, ko-3, and ko-11) via Sanger sequencing.

Previous studies indicated that *SlERF.J2* was highly expressed in tomato fruits and leaves, suggesting its involvement in leaf development and fruit ripening^[37]^. To uncover transcriptional changes in *SlERF.J2* in response to abiotic stimuli, we quantified its relative expression in tomato samples sampled at multiple time points following different stress treatments. As shown in Fig. 2a, expression of *SlERF.J2* was strongly inhibited by drought stress 4–8 h after treatment, but were restored to normal levels by 24 h. As depicted in Fig. 2b, the expression of *SlERF.J2* remained steady within the initial 12 h of salt stress, yet declined markedly at 24 h. Collectively, these results demonstrate that *SlERF.J2* is rapidly activated in the early stress stage and initiates early defense responses in tomato via transcriptional modulation of the downstream stress-associated genes. Under 4 °C cold treatment (Fig. 2c), the expression of *SlERF.J2* was suppressed at 2–12 h but was sharply upregulated at 24 h. Under 42 °C heat stress (Fig. 2d), the expression of *SlERF.J2* showed a gradual downward trend and decreased at 24 h. Collectively, these observations suggest that *SlERF.J2* mediates broad-spectrum abiotic stress tolerance in tomato plants.

To explore hormonal responses, WT seedlings were treated with IAA, GA_3_, and MeJA, and the expression of *SlERF.J2* was recorded at different time points. The data revealed that *SlERF.J2* transcript levels decreased drastically at 2 h and persisted at low levels throughout the subsequent time points (Fig. 2e–g). This indicates that *SlERF.J2* may function early in hormone signaling pathways in tomato.

### Knockout of *SlERF.J2* increases tolerance to PEG6000 treatment

To explore *SlERF.J2* functions, we acquired three knockout lines: *slerf.j2ko*-1 with a 1-bp insertion, *slerf.j2ko*-3 with a 7-bp deletion, and *slerf.j2ko*-11 carrying a 2-bp deletion and a 1-bp substitution on two alleles, respectively (Fig. 2h–j). All the abovementioned mutations were located within the first exon of the *SlERF.J2* gene, leading to frameshift and premature termination of translation.

**Figure 3:**
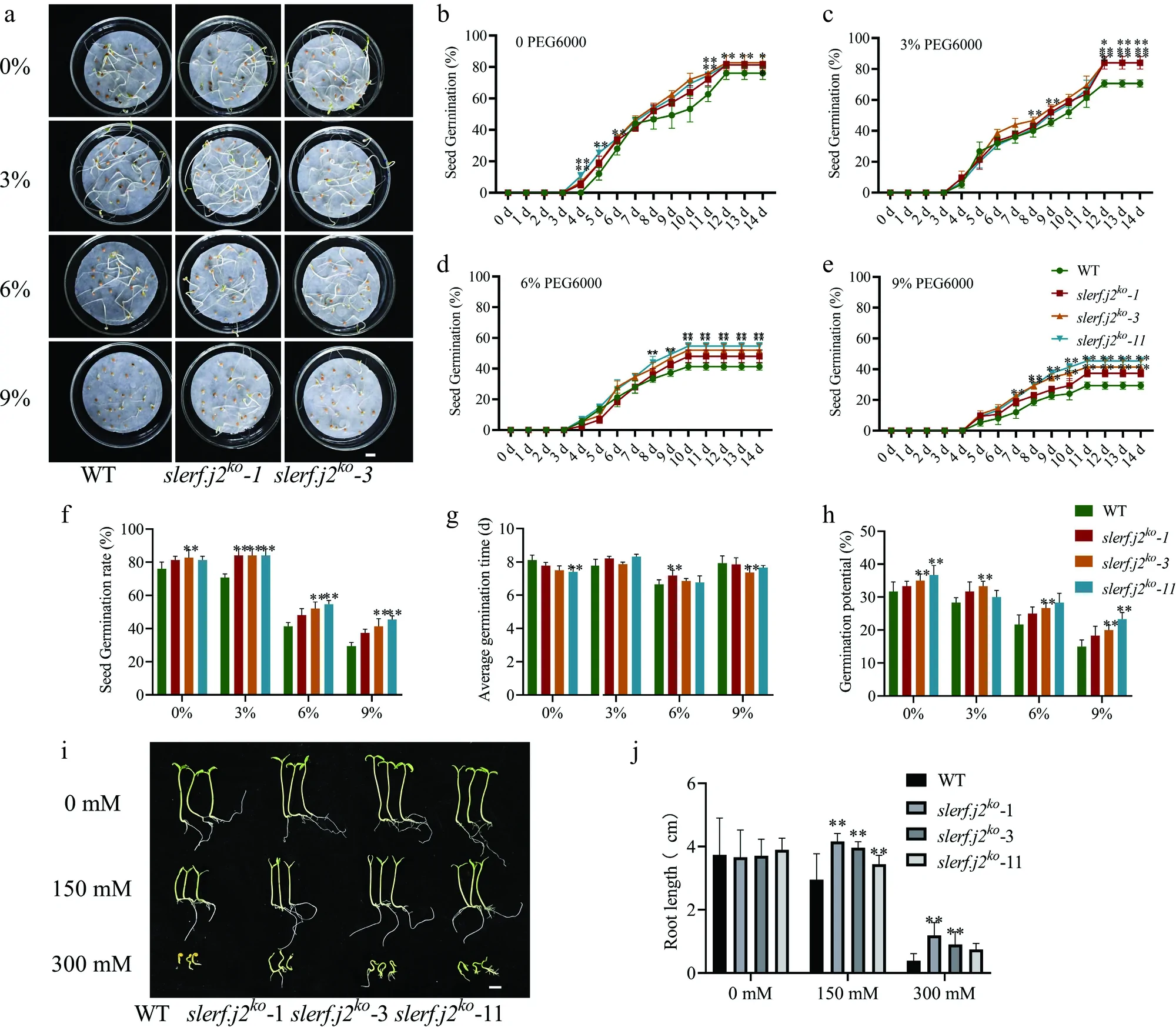
Loss-of-function *slerf.j2* mutants gained increased tolerance to PEG6000 and mannitol-induced osmotic stress during seed germination and post-germination seedling establishment. (a) Phenotypic analysis of seed germination in the WT and *slerf.j2* knockout lines under varying PEG6000 stress levels. The images display the germination status of seeds in Petri dishes under different drought-simulating osmotic stress conditions. (b)–(e) Profiles of the germination rate over time of WT and *slerf.j2*^*ko*^ lines under the (b) 0%, (c) 3%, (d) 6%, and (e) 9% PEG6000 treatment, respectively; data reflect the percentage of germinated seeds over time. (f) Statistical evaluation of the ultimate seed germination levels of the tested lines under graded PEG6000 stress conditions. (g) Average germination time of the WT and knockout lines under different PEG6000 treatments. (h) Germination potential of WT and knockout lines under different PEG6000 concentrations. Growth status of WT and knockout lines seedlings (*n* ≥ 25) cultured on MS media containing a gradient of PEG6000 concentrations (0%, 3%, 6%, 9%) for 5 days after germination to observe the phenotypic growth traits. (i) Seedling phenotypes of WT and knockout lines under treatment with different concentrations of mannitol (*n* ≥ 25). Scale bar = 1 cm. (j) Root length of WT and knockout materials determined along a mannitol concentration gradient. Student's *t*-test was used to assess statistical differences relative to the WT, with * *p* < 0.05 and ** *p* < 0.01 as the significance thresholds.

PEG6000 induces osmotic stress in plants to mimic drought stress^[40]^. Here, WT and *slerf.j2*^*ko*^ seeds were treated with a gradient of PEG6000 concentrations, and germination indexes were determined to clarify *SlERF.J2'*s functions in the germination of tomato seeds. The results in Fig. 3a showed that the *slerf.j2* knockout (*slerf.j2*^*ko*^) lines exhibited more vigorous seed germination under the 3% and 6% PEG6000 treatments. Under the 9% PEG6000 treatment, the germination density of all lines decreased, but the *slerf.j2*^*ko*^ lines still performed slightly better than the WT. The results in Fig. 3b–e indicate that under the 3% and 6% PEG6000 treatments, the *slerf.j2*^*ko*^ lines initiated germination earlier with higher rates, and their germination curves stabilized 2**–**3 d ahead of those of the WT. Under the 9% PEG6000 treatment, seed germination was delayed in all lines, but the *slerf.j2*^*ko*^ lines maintained slightly higher germination levels throughout the entire germination period.

As shown in Fig. 3f, the WT and *slerf.j2* knockout lines exhibited similar germination percentages under normal conditions, both reaching approximately 80%. Under the 3% PEG6000 treatment, *slerf.j2* knockout lines exhibited germination rates between 77% and 85%, whereas that of the WT was only 45%. Under the 6% PEG6000 treatment, the germination rates of the *slerf.j2*^*ko*^ lines remained between 66% and 72%, whereas that of the WT dropped to 39%. Under the 9% PEG6000 treatment, the *slerf.j2*^*ko*^ lines still exhibited a slight superiority in its germination rate over the WT. Knockout of *SlERF.J2* enhances the germination performance of tomato seeds under PEG6000-simulated drought stress, particularly under moderate stress conditions (3% and 6% PEG6000), as evidenced by the higher germination rate, better germination uniformity, and stronger germination vigor.

### Loss of *SlERF.J2* function improves the osmotic tolerance of tomato seedlings under mannitol stress

Mannitol treatments were applied to tomato seedlings to explore the role of *SlERF.J2* in osmotic stress adaptation. After 5 d of treatment, we characterized the seedlings' morphology and quantitatively analyzed their root lengths (Fig. 3). As shown in Fig. 3i, the differences in growth between the WT and *slerf.j2*^*ko*^ lines are stress-dependent.

Root length is a core indicator of seedlings' stress tolerance. As illustrated in Fig. 3j, WT and *slerf.j2* knockout seedlings exhibited similar root lengths under 0 mM mannitol conditions. Under 150 mM mannitol, all *slerf.j2* knockout lines possessed significantly longer roots compared with the WT. The 300 mM mannitol treatment almost completely blocked root growth in WT plants, although the knockout lines retained substantial root elongation capacity. Taken together, these observations reveal that functional deletion of *SlERF.J2* enhances tomato seedlings' resistance against mannitol-induced osmotic stress. Under moderate and severe osmotic stress conditions, the root growth of *slerf.j2*^*ko*^ lines was less inhibited.

### The *SlERF.J2* loss-of-function mutation promotes tolerance to saline stress during seed germination and seedling development in tomato

**Figure 4:**
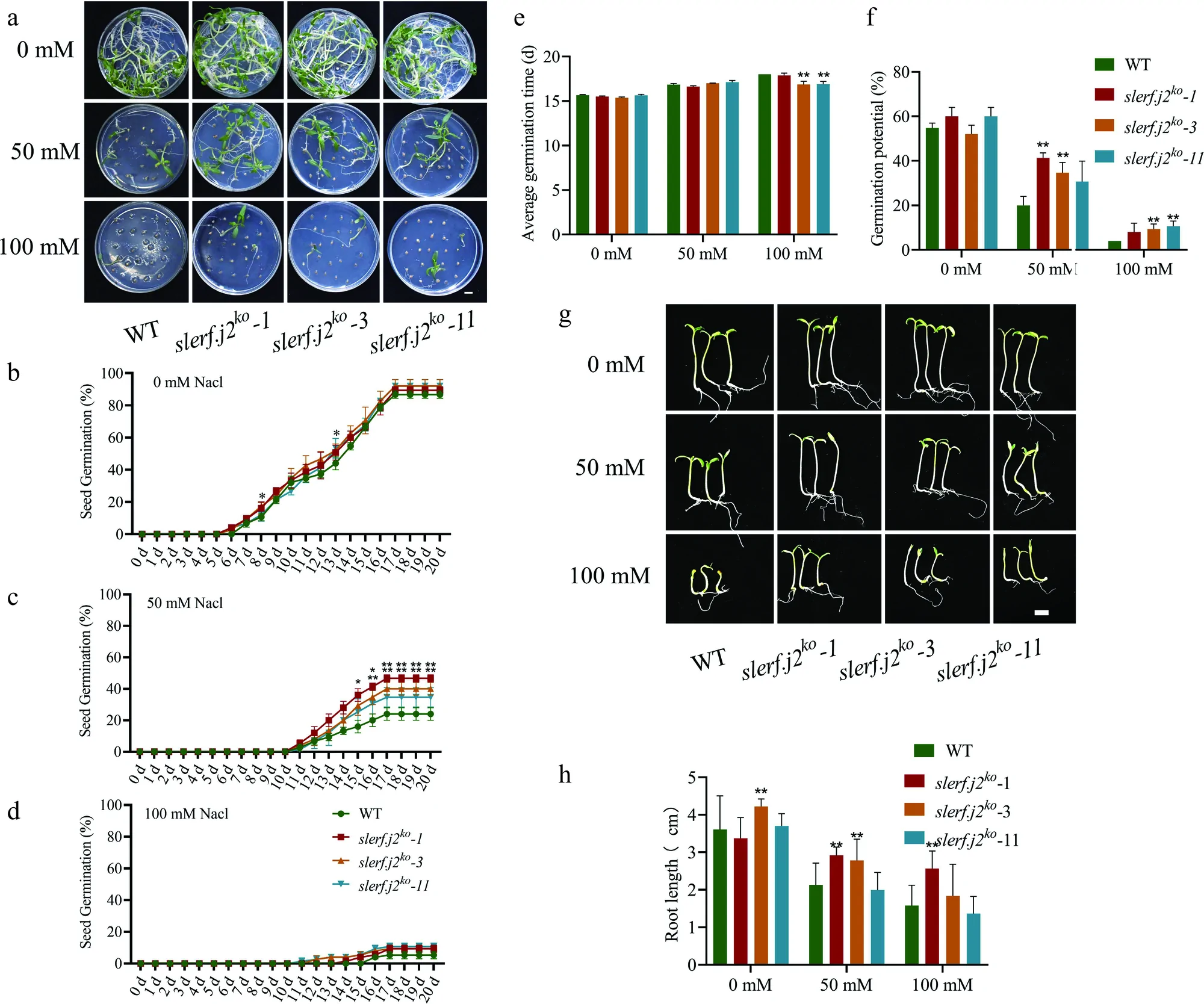
The *slerf.j2* mutants exhibited superior germination capacity and seedling growth under salt stress conditions. (a) Germination performance of WT and *slerf.j2* knockout tomato lines (ko-1, ko-3, and ko-11) under gradient NaCl treatments (0, 50, and 100 mM). The images show the germination status of seeds in Petri dishes under different salt concentrations. (b)–(d) Germination rate curves across time for the genotypes under three NaCl concentrations: (b) 0 mM, (c) 50 mM, and (d) 100 mM; vertical data denote cumulative seed germination percentages at different time points. (e) Statistical analysis of the average germination time of WT and *slerf.j2*^*ko*^ lines under different NaCl concentrations. (f) A comparison of germination potential between WT plants and *slerf.j2* knockout lines under varying salinity treatments. (g) Seedling phenotypes of WT and *slerf.j2*^*ko*^ lines (ko-1, ko-3, ko-11) treated with different concentrations of NaCl. The images display the root morphology of seedlings under different salt concentrations (scale bar = 1 cm). (h) Quantification of root length in WT and *slerf.j2* mutant lines exposed to a gradient of NaCl treatments. Seedling growth of both lines (*n* ≥ 25) cultured on NaCl-supplemented MS medium for 5 d. Data are the mean ± standard error (SE). Statistical analysis was performed via Student's *t*-test; asterisks denote significant differences relative to the WT (* *p* < 0.05, ** *p* < 0.01).

To clarify the regulatory function of *SlERF.J2* during seed germination under salt stress, tomato seeds were subjected to NaCl solutions with varying concentration gradients (Fig. 4). As shown in Fig. 4a, under 100 mM NaCl, WT seeds barely germinated, whereas the *slerf.j2*^*ko*^ lines maintained detectable germination levels. Under 0 mM NaCl (Fig. 4b), the *slerf.j2*^*ko*^ lines initiated germination earlier than the WT, though all lines achieved comparable final maximum germination rates. Under 50 mM NaCl (Fig. 4c), the *slerf.j2* mutants showed consistently higher germination rates than the WT. Under 100 mM NaCl (Fig. 4d), overall germination was drastically reduced across all lines, but the *slerf.j2*^*ko*^ lines still maintained slightly higher germination levels than the WT.

Under normal conditions, the average germination time and potential showed no obvious differences between the WT and *slerf.j2* knockout lines. By contrast, 50 and 100 mM NaCl delayed seed germination in the WT, prolonged the average germination time, and reduced germination potential. Collectively, these results demonstrate that knockout of *SlERF.J2* enhances tomato seeds' germination tolerance to NaCl-induced salt stress. Specifically, under moderate to severe salt stress conditions, *slerf.j2*^*ko*^ lines exhibited higher germination rates, faster germination speed, and stronger germination vigor.

To elucidate how *SlERF.J2* modulates tomato seedlings' growth under salinity, we treated WT and *slerf.j2*^*ko*^ seedlings with NaCl. After 5 d of treatment, we characterized the seedlings' morphology and quantified their root lengths (Fig. 4). As shown in Fig. 4g, exposure to 50 mmol/L NaCl markedly suppressed the growth of WT seedlings, whereas the *slerf.j2* knockout lines exhibited robust growth. Under the 100 mmol/L NaCl treatment, WT seedlings almost ceased growth, whereas the *slerf.j2*^*ko*^ lines still exhibited a certain degree of root elongation. As shown in Fig. 4h, under 0 mmol/L NaCl, only the *slerf.j2*^*ko*^*-*1 line exhibited significantly longer roots than the WT. By contrast, the remaining *slerf.j2*^*ko*^ mutants exhibited negligible phenotypic discrepancies compared with WT plants. In addition, under the 50 and 100 mmol/L NaCl stress treatments, the root growth of all lines was inhibited, but the *slerf.j2*^*ko*^ lines still showed significantly longer roots than the WT. Collectively, these results reveal that the knockout of *SlERF.J2* improves salt stress tolerance in tomato. The root development of *slerf.j2*^*ko*^ plants was subjected to milder growth suppression under moderate and high-salinity conditions, indicative of superior salt tolerance.

### *SlERF.J2* knockout confers enhanced salt and drought tolerance in tomato

**Figure 5:**
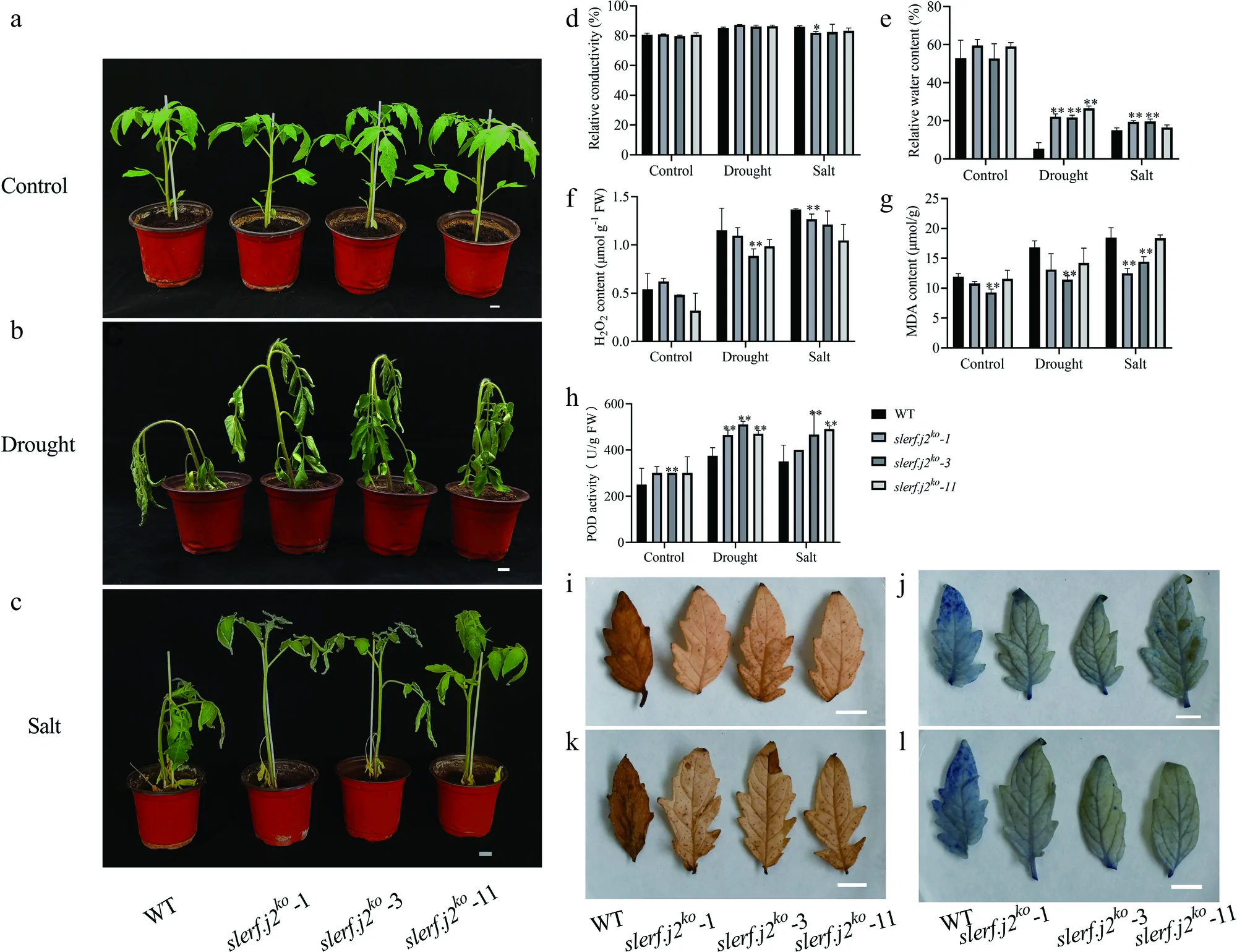
The *slerf.j2*^*ko*^ lines show improved growth performance and altered physiological responses under drought and salt stress. (a)–(c) Phenotypic traits of WT and *slerf.j2*^*ko*^ lines (ko-1, ko-3, ko-11) under (a) control, (b) drought, and (c) salt stress conditions. Each treatment consisted of at least 15 tomato seedlings. Under drought and salt stress, WT plants wilted severely with inhibited growth, whereas *slerf.j2*^*ko*^ lines maintained relatively robust growth. Scale bars in (a–c) = 1 cm. (d)–(h) Comparisons of REC, RWC, H_2_O_2_ content, MDA content, and POD activity in WT and *slerf.j2*^*ko*^ lines. The results are presented as the mean ± SE based on three biological repeats, with significance compared with the WT at * *p* < 0.05 and ** *p* < 0.01 levels. (i), (k) DAB staining of leaves after 15 days of salt treatment and 21 days of drought treatment. Brown deposits indicate H_2_O_2_ accumulation. (j), (l) Trypan blue staining under stress conditions. Dark blue denotes severe cell death; lighter staining shows less cell damage in the mutants. Scale bars in (i)–(l) = 1 cm.

To explore *SlERF.J2*'s roles in adaptation to abiotic stress, tomato seedlings were exposed to drought and salt treatments, followed by phenotypic observation and calculation of the stress-related physiological index (Fig. 5).

As presented in Fig. 5a, all tested genotypes grew robustly without distinct morphological disparities in the absence of stress. Under drought stress (Fig. 5b), WT plants showed severe wilting, whereas *slerf.j2*^*ko*^ lines retained relatively turgid leaves with milder wilting. Under salt stress (Fig. 5c), WT plants displayed stunted growth and extensive leaf necrosis, whereas *slerf.j2*^*ko*^ lines maintained a better growth status.

Relative electrical conductivity serves as an indicator of cell membrane damage. No phenotypic differences existed between genotypes under normal conditions (Fig. 5d). Upon drought and salt stress exposure, WT plants exhibited marginally higher REC than *slerf.j2*^*ko*^ lines, indicating more severe cell membrane injury in WT seedlings. For RWC (Fig. 5e), under control conditions, all lines had comparable RWC. Under stress, WT plants showed a drastic reduction in RWC (about 5% under drought; about 10% under salt), whereas *slerf.j2*^*ko*^ lines maintained significantly higher water content, reflecting stronger water retention capacity. For H_2_O_2_ levels (Fig. 5f), under control conditions, H_2_O_2_ levels were similar across genotypes. Under stress conditions, WT plants accumulated significantly more H_2_O_2_ than *slerf.j2*^*ko*^ lines, suggesting lower ROS accumulation in *slerf.j2*^*ko*^ lines under stress. MDA is a marker of membrane lipid peroxidation. No significant differences were found between the two genotypes under control conditions. Under stress conditions, MDA content was significantly elevated in WT plants relative to *slerf.j2* lines, confirming less membrane lipid peroxidation in *slerf.j2*^*ko*^ lines (Fig. 5g). POD is a key antioxidant enzyme. Under drought and salt stress, *slerf.j2* mutants had higher POD activity than WT plants, indicating stronger antioxidant defense capacity in *slerf.j2*^*ko*^ lines (Fig. 5h). Collectively, these findings confirm that *SlERF.J2* knockout strengthens tomato's tolerance to drought and salinity stresses. Under stress conditions, *slerf.j2*^*ko*^ lines exhibit milder phenotypic damage, less cell membrane injury, stronger water retention capacity, lower ROS accumulation, and higher antioxidant enzyme activity.

### Knockout of *slerf.j2* alleviates leaf damage and reduces cell death under stress conditions in tomato

To further assess stress-induced damage in tomato leaves, we ran staining assays using DAB. Specifically, POD can mediate the reaction between H_2_O_2_ and DAB, producing an insoluble brown product, and DAB staining enables the visualization of the spatial distribution of H_2_O_2_ in leaf tissues.

The DAB staining results showed that salt stress caused severe leaf browning in WT plants (Fig. 5i). In contrast, the leaves of *slerf.j2*^*ko*^ lines exhibited only mild chlorosis and sporadic necrotic spots, indicating that knockout of this gene effectively alleviates salt stress-induced damage in tomato plants. Under drought stress (Fig. 5k), WT leaves suffered extensive browning and tissue collapse, whereas the *slerf.j2*^*ko*^ lines maintained a relatively intact leaf morphology with markedly reduced necrosis. Trypan blue staining was adopted to visualize cellular damage in the leaves for evaluating stress-triggered cell death. (Fig. 5j,l). The results demonstrated that under both salt and drought stresses, WT leaves displayed intense blue staining signals, indicative of widespread cell death, whereas the staining signals of *slerf.j2*^*ko*^ lines were significantly weakened, which was consistent with the phenotypic observations. Collectively, these results indicate that *SlERF.J2* impairs tomato's resistance to salinity and drought by accelerating stress-mediated leaf cell death.

### Relative expression of stress-associated genes between WT and *slerf.j2*^*ko*^ lines under abiotic stress

qRT-PCR analysis was used to quantify the transcription of stress-responsive genes in WT and mutant plants under normal, drought, and salt treatments, aiming to unravel the regulatory mechanism of *SlERF.J2*.

**Figure 6:**
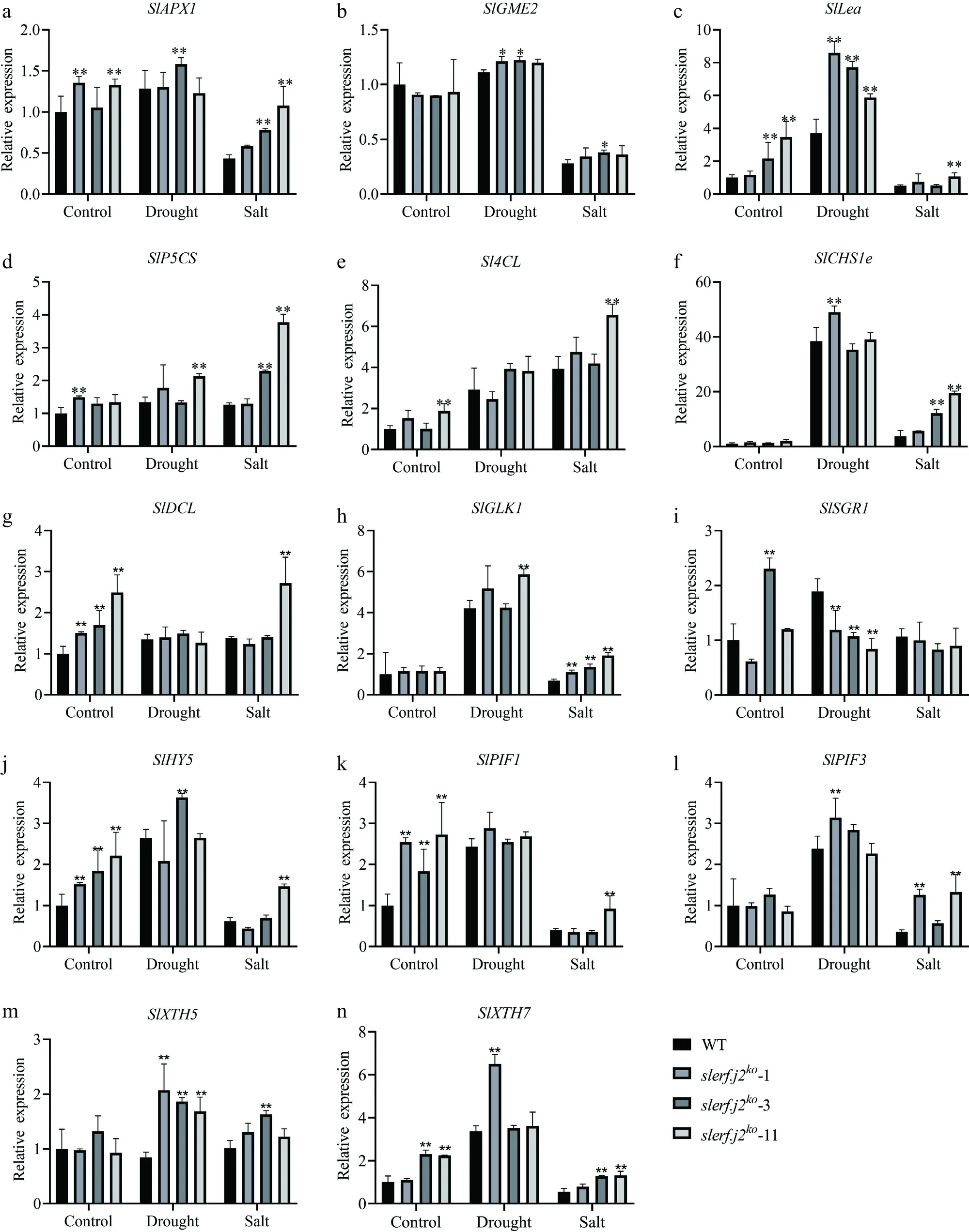
Stress-related gene expression in *slerf.j2* mutants under normal, drought, and salt conditions. (a)–(n) Transcript abundance of 14 stress-inducible genes: (a) *SlAPX1*, (b) *SlGME2*, (c) *SlLea*, (d) *SlP5CS*, (e) *Sl4CL*, (f) *SlCHS1e*, (g) *SlDCL*, (h) *SlGLK1*, (i) *SlSGR1*, (j) *SlHY5*, (k) *SlPIF1*, (l) *SlPIF3*, (m) *SlXTH5*, and (n) *SlXTH7* in WT and *slerf.j2*^*ko*^ plants under stress treatments. The results are expressed as mean ± standard error obtained from three biological repeats; Student’s *t*-test was applied for analyzing significance (* *p* < 0.05, ** *p* < 0.01).

Under stress conditions, multiple stress-related genes (*SlAPX1*, *SlGME2*, *SlLea*, *SlP5CS*, *Sl4CL*, *SlCHS1e*, *SlDCL*, *SlGLK1*, *SlSGR1*, *SlHY5*, *SlPIF1*, *SlPIF3*, *SlXTH5*, and *SlXTH7*) displayed significantly increased transcription in *slerf.j2* knockout lines than in the WT, albeitwith minor downregulation of a few genes in *slerf.j2*^*ko*^ lines under salt stress relative to their levels under drought, though still higher than in most WT samples (Fig. 6). Among these genes, *SlAPX1* is related to antioxidant defense. *SlGME2* is a key ascorbic acid (AsA) synthetase gene^[41]^. *SlGLK1* is related to chloroplast function and development. *SlLea* is related to stress responses^[42]^. *SlP5CS* is a key proline biosynthesis (osmotic adjustment) gene^[43]^. *Sl4CL*^[44]^ and *SlCHS1e*^[45]^ are related to carotenoid and flavonoid biosynthesis. *SlDCL* is related to stress signal transduction. *SlHY5*, *SlPIF1*, and *SlPIF3* are related to light and stress signal. *SlXTH5* and *SlXTH7* are related to secondary metabolism. Collectively, *SlERF.J2* knockout upregulates stress-, metabolism- and chloroplast-related genes, confirming its negative regulatory role in abiotic stress tolerance.

### Expression of phytohormone signaling-related genes in *slerf.j2* mutants under stress

**Figure 7:**
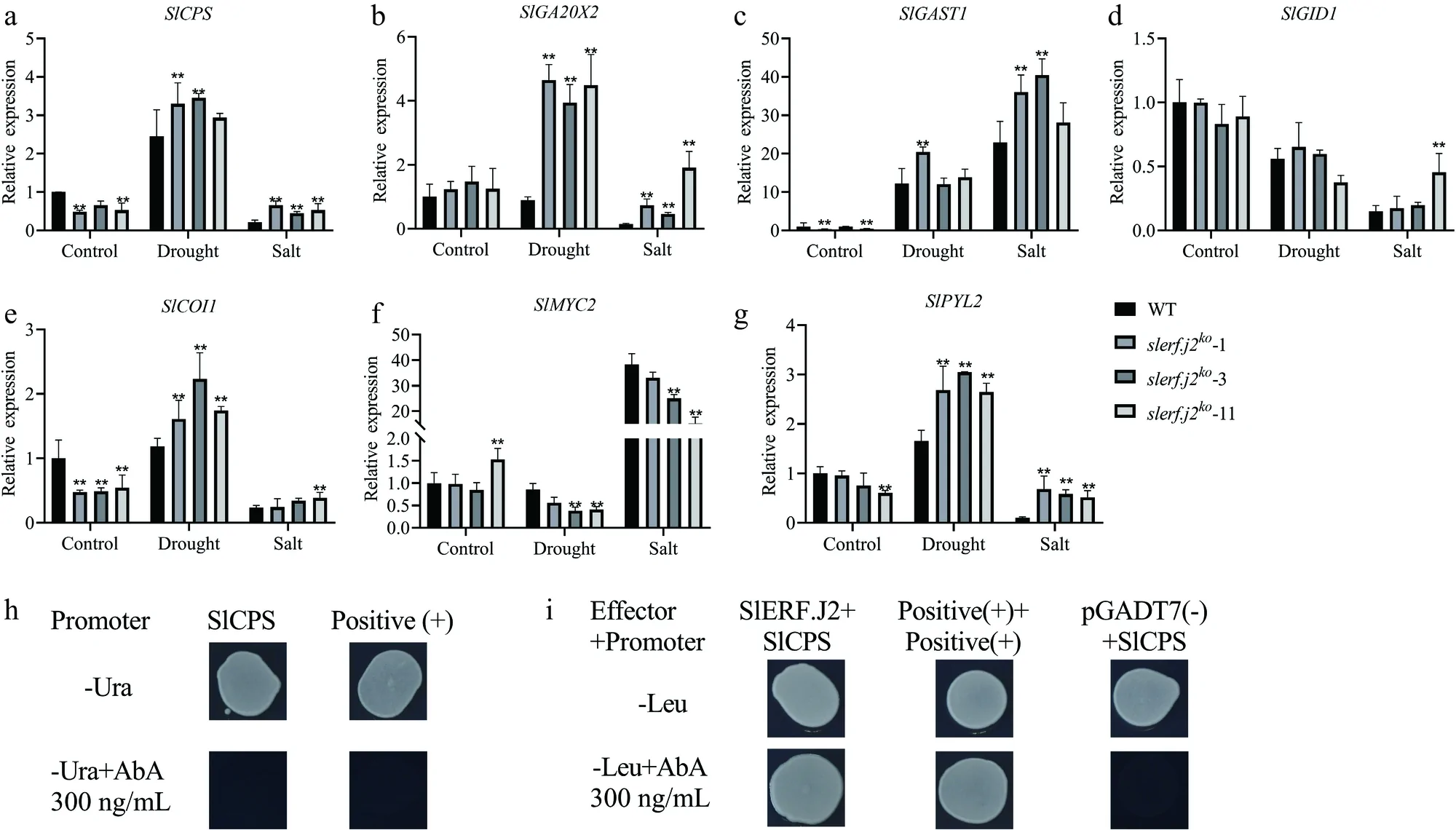
Transcript profiles of stress-inducible genes in *slerf.j2*^*ko*^ tomato plants across control, drought, and salinity treatments. Y1H assays validated that SlERF.J2 targets the *SlCPS* promoter. (a)–(g) Expression of seven hormone-associated genes in WT and *slerf.j2* mutant lines: (a) *SlCPS*; (b) *SlGA20x2*; (c) *SlGAST1*; (d) *SlGID1*; (e) *SlCOI1*; (f) *SlMYC2*; (g) *SlPYL2* (* *p* < 0.05, ** *p* < 0.01). (h) Autoactivation detection of the *SlCPS* promoter bait vector. Yeast cells harboring the *SlCPS* promoter bait plasmid were cultured on –Ura and –Ura + 300 ng/mL ABA plates. The *SlCPS* promoter bait did not support yeast growth on –Ura + ABA plates, demonstrating no autoactivation activity. (i) Y1H interaction assay between SlERF.J2 and the *SlCPS* promoter. Yeast cells co-transformed with the SlERF.J2 prey vector + the *SlCPS* promoter bait vector were cultured on –Leu and –Leu + 300 ng/mL ABA plates. Growth on –Leu + ABA plates verified the direct interaction of SlERF.J2 with the *SlCPS* promoter. The absence of growth in the pGADT7 + *SlCPS* group confirms the interaction's specificity.

To explore the function of *SlERF.J2* in abiotic stress, the expression levels of hormone-related genes were analyzed (Fig. 7a–g). After drought treatment, most hormone-related genes were markedly upregulated in *slerf.j2* mutants, wherease the expression of *SlGID1* slightly decreased. After salt treatment, all these genes (including *SlGID1*) exhibited notably higher transcription levels in *slerf.j2*^*ko*^ lines relative to WT plants (with some genes showing over 20-fold elevation). Among these genes, *SlCPS* and *SlGA20ox2* are core functional enzymes in the gibberellin biosynthesis pathway. *SlGAST1* is involved in gibberellin signal-mediated growth regulation. It enhances plants' stress tolerance by participating in cellular antioxidant defense and regulating stress-related metabolic processes. *SlCOI1* and *SlMYC2* are core JA signaling components. *SlPYL2* is an ABA signaling receptor. These results imply that *SlERF.J2* may regulate abiotic stress adaptation in tomato by modulating the transcription of genes in various phytohormone signaling pathways.

### Validation of the direct binding between SlERF.J2 and the *SlCPS* promoter via the Y1H assay

Since *SlERF.J2* knockout enhances abiotic stress tolerance in tomato, we analyzed promoter sequences of differentially expressed genes. Analysis demonstrated that the promoter of the *SlCPS* gene contains ERF-binding elements (Supplementary Fig. S1). The Y1H assay verified SlERF.J2’s binding to the *SlCPS* promoter (Fig. 7h, i). Self-activation verification revealed yeast carrying *SlCPS* promoter grew on –Ura medium, yet failed on the medium with 300 ng/mL ABA, showing no self-activation activity. Subsequently, the binding assay results (Fig. 7i) revealed that yeast co-transformed with SlERF.J2 and the *SlCPS* promoter grew well on –Leu medium containing 300 ng/mL ABA, whereas the control group showed no growth. *In vivo* assays confirmed that *SlERF.J2* specifically binds to the *SlCPS* promoter. As another AP2/ERF family member, *SlDREB* targets DRE/CRT (Dehydration-Responsive Element/C-repeat responsive element) motifs in this promoter and positively regulates drought resistance by regulating plant morphology^[46]^. Collectively, we propose that SlERF.J2 modulates tomato's stress responses through regulating the expression of *SlCPS*.

## Discussion

AP2/ERF proteins feature versatile biological functions and are indispensable for plants' developmental processes and tolerance of environmental challenges. Among the unfavorable abiotic factors, drought and salt severely restrict plant growth; accordingly, plants evolve elaborate multilevel adaptive pathways at the molecular, physiological, and biochemical levels. Transgenic *A. thaliana* with suppressed *AtERF5* activity showed markedly improved salt tolerance^[24]^. Suppression of *AtERF13* and *AtERF6* elevates cadmium resistance in *A. thaliana*^[47]^. GhJAZ3 interacts with GhWRKY33 and negatively regulates plants' resistance to *Verticillium dahliae* by inhibiting the expression of *AtERF1* and *GhERF2*^[48]^. Furthermore, the aforementioned ERF family genes in *A. thaliana* share high homology with *SlERF.J2*, indicating that *SlERF.J2* potentially participates in the modulation of plants' stress tolerance. We systematically investigated how *SlERF.J2* participates in drought and salt stress tolerance in tomato.

Most identified tomato ERF transcription factors. including *SlERF1*, *SlERF8*, *SlERF15*, and *SlERF-B3*, positively regulate abiotic stress resistance. Nevertheless, only a small subset of ERF members were documented as negative modulators of stress resistance within tomato. Here, we show that *SlERF.J2* negatively modulates stress tolerance in tomato by inhibiting the ABA signaling pathway and stomatal closure, which is functionally distinct from most known stress-positive tomato ERFs. *SlERF.J2* has been newly characterized as a stress-suppressive ERF, broadening or understanding of functional diversification within the tomato AP2/ERF family against abiotic challenges.

Abiotic stress tolerance is a multifaceted trait manifesting at distinct growth phases, and our results demonstrate that *SlERF.J2* knockout improves performance at each stage. Under the drought-mimetic PEG6000 treatment, *slerf.j2*^*ko*^ lines exhibited higher germination rates, shorter germination times, and stronger germination potential than the WT (Fig. 3), consistent with the link between enhanced seed germination under osmotic stress and drought tolerance^[49]^. Similarly, under salt stress, *slerf.j2*^*ko*^ seeds germinated more efficiently, and the seedlings showed longer roots (Fig. 4), which are traits that reflect reduced stress-induced growth inhibition. Under drought and salt conditions, mature *slerf.j2* knockout lines presented milder wilting, higher RWC, and lower electrolyte leakage (Fig. 5). These traits reflected superior cell stability and water retention capacity, which are typical characteristics of stress-resistant plants^[50]^. Uniform phenotypic variations observed across multiple developmental stages corroborate that *SlERF.J2* acts as a negative modulator governing tomato's tolerance of drought and salinity.

Environmental stresses induce ROS accumulation in plants. Excessive ROS accumulation leads to lipid peroxidation and cellular injury, which consequently impairs plant growth and crop productivity^[51−52]^. In this study, under drought and salt stresses, the *slerf.j2*^*ko*^ lines accumulated lower levels of H_2_O_2_ and MDA but exhibited higher POD activity (Fig. 5). As a marker of lipid peroxidation, reduced MDA levels directly indicate that knockout of *SlERF.J2* can effectively alleviate the cell membrane damage caused by excessive ROS accumulation. The increase in POD activity is only one aspect of its regulation of the antioxidant defense system, not the sole pathway. DAB staining results corroborated this conclusion, revealing less H_2_O_2_ accumulation in the leaves of mutant lines. Loss of *SlERF.J2*'s function activates plant antioxidant systems, alleviating ROS-caused cell injury. Trypan blue staining, a technique used to visualize dead cells^[25]^, further confirmed that mutant lines exhibited less severe cellular damage in the leaves. This finding is consistent with reduced H_2_O_2_ accumulation, indicating that after the knockout of *SlERF.J2*, the ROS scavenging capacity of the plant is comprehensively improved, rather than relying solely on the regulation of POD activity alone. Additionally, *SlP5CS* (a key gene for proline biosynthesis, a major osmolyte) was significantly upregulated in *slerf.j2*^*ko*^ lines. Elevated *SlP5CS* expression likely promotes proline accumulation, which maintains cellular turgor and protects macromolecules under osmotic stress^[53]^. Together, these physiological changes explain the enhanced stress resilience of *slerf.j2*^*ko*^ plants.

As a transcription factor, *SlERF.J2* probably regulates stress responses via its downstream target genes. Under stress, multiple functional genes were markedly upregulated in *slerf.j2* mutants, covering ROS-scavenging antioxidant genes (*SlAPX1*, *SlGME2*), drought-resistant protective genes (*SlLea*), osmotic adjustment-related genes (*SlP5CS*), and key genes responsible for secondary metabolism and stress signal transduction (*Sl4CL*, *SlCHSle*). Stress treatment induced prominent upregulation of genes linked to ROS clearance, cell stabilization, osmotic adjustment, secondary metabolism, and stress signaling in *slerf.j2* knockout lines, demonstrating the involvement of *SlERF.J2* in stress regulation by targeting these genes.

Hormone crosstalk is a central process for integrating stress signals and optimizing plants' stress responses. Our results demonstrated that *SlERF.J2* participates in regulating the ABA, GA, and JA signaling pathways. *SlPYL2*, which encodes an ABA receptor, initiates ABA-mediated stress responses (stomatal closure, osmolyte accumulation) and was significantly upregulated in *slerf.j2*^*ko*^ lines under drought stress. This suggests that *SlERF.J2* represses ABA signaling under stress. Knockout of *SlERF.J2* enhances plants' ABA perception, thereby regulating stomatal movement and promoting physiological adjustments associated with stress responses. Genes involved in GA biosynthesis (*SlGA20ox2*) and perception (*SlGID1*) exhibited altered expression in *slerf.j2*^*ko*^ lines under salt stress. GAs typically promote growth, but their signaling is fine-tuned under stress to prioritize defense over growth. The upregulation of *SlGA20ox2* in *slerf.j2*^ko^ lines may reflect a balanced regulation, in which *SlERF.J2* knockout likely relieves the repression of GA-related genes and allows plants to maintain moderate growth capacity while sustaining stress defense. *SlCOI1* (a JA receptor) and *SlMYC2* (a JA-responsive transcription factor), which are key components of JA-mediated stress and defense responses, were upregulated in *slerf.j2*^ko^ lines under stress. Knockout of this gene enhances JA-mediated defense, which synergizes with ABA signaling to improve stress resistance.

**Figure 8:**
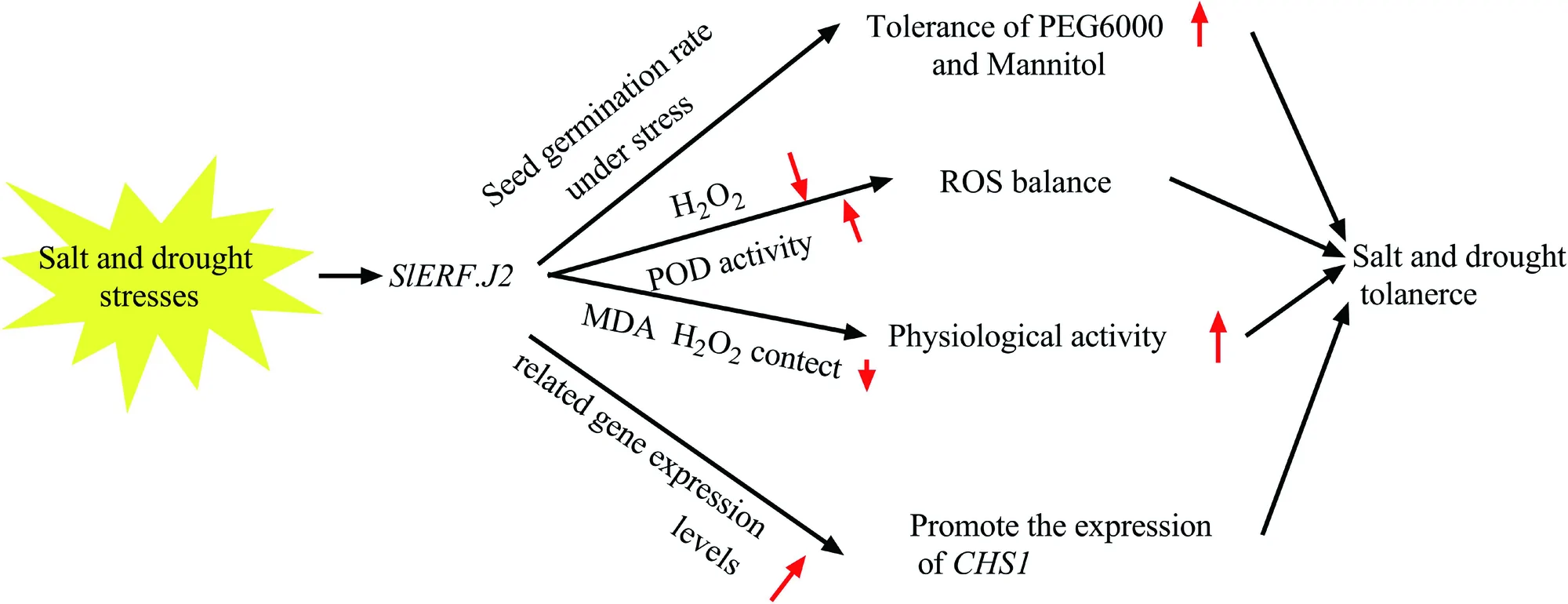
Mechanistic model of *SlERF.J2*-mediated regulation of salt and drought tolerance in tomato.

Furthermore, *SlCPS* acts as a pivotal gene in the GA biosynthesis pathway. It modulates several essential developmental processes in tomatoes by governing the levels of bioactive GAs^[54,55]^. *SlDREB* represses the transcription of core genes responsible for GA biosynthesis, such as *SlCPS* and *SlGA20ox1*, *SlGA20ox2*, and *SlGA20ox4*. It specifically targets DRE/CRT elements in the *SlCPS* promoter, and enhances drought resistance by restraining leaf growth and internode elongation^[46]^. After stress treatment, *SlCPS* expression differed greatly between knockout mutants and WT tomatoes. Moreover, SlERF.J2 bound the *SlCPS* promoter. This suggests that knocking out *SlERF.J2* improves tomato's drought and salt resistance by modulating the gibberellin synthesis pathways (Fig. 8).

The AP2/ERF family is large in size and diverse in function. Most identified members such as *OsERF19* and *AtERF71* serve as positive regulators of stress tolerance, whereas several members, including *ERF012*, function as negative regulators. This study identifies *SlERF.J2* as a promising target for improving abiotic stress tolerance in tomato. The *slerf.j2*^*ko*^ lines exhibit enhanced drought and salt resistance across multiple developmental stages, providing valuable genetic resources for molecular breeding. Future research will further identify additional direct target genes of *SlERF.J2*, explore its crosstalk with hormone signaling pathways (ABA, ethylene), and validate its field performance to facilitate agricultural application.

## Conclusions

This study systematically elucidated the role of *SlERF.J2* in regulating salt and drought resistance in tomato. Our results demonstrated that knockout of *SlERF.J2* significantly improved seed germination performance under PEG6000-induced osmotic stress and enhanced seedlings' growth tolerance under mannitol treatment. Knockout of *SlERF.J2* rebalanced cellular ROS homeostasis by reducing H_2_O_2_ accumulation and increasing POD activity, thereby alleviating oxidative damage and modulating plants' stress adaptation. In addition, the data verify the inhibitory effect of *SlERF.J2* on the expression of stress- and hormone-related genes, which, in turn, determine tomato's resistance to adverse abiotic environments. This work identifies *SlERF.J2* as a key negative regulator in plants' stress adaptation. Through bioengineering of the ERF *SlERF.J2*, drought- and salt stress-tolerant tomato germplasm was created.

## SUPPLEMENTARY DATA

Supplementary data to this article can be found online.

## Data Availability

All experimental results and data from this study are provided in the main text and online supplementary materials.
