# Supplementary material for: Generating drought- and salt-tolerant tomato germplasm through bioengineering of the ethylene response factor SlERF.J2
Source: Eng Life Sci. 2026 Jun 27;26(5-6):e003. doi: 10.48130/els-0026-0003 (PMC13599428; doi:10.48130/els-0026-0003)
Supplement: Supplementary file 1 [file els-2026-26-5-6-0003-S1.zip › 10.48130_els-0026-0003-Suppl-TableS1.pdf]

Supplementary Table S1. Details of Primers for qRT-PCR Amplification

Primer names	Primer sequence
q*SlERF.J2*-F	TATACTGCTAGGGCGGTCAATG
q*SlERF.J2*-R	AAGCCGAACATATCCATTTGACA
q*SlCAC*-F	CCTCCGTTGTGATGTAACTGG
q*SlCAC*-R	ATTGGTGGAAAGTAACATCATCG
q*SlEF1α*-F	TACTGGTGGTTTTGAAGCTG
q*SlEF1α-R*	AACTTCCTTCACGATTTCATCATA
q*SlApx1*-F	TCAGTGATCCTGCTTTCCGC
q*SlApx1*-R	TGTCACCACCCTCCCAACTCT
q*SlGME2*-F	CCATCACATTCCAGGACCAGA
q*SlGME2*-R	CGTAATCCTCAACCCATCCTTC
q*SlLea*-F	TATTGGTAAAGATTGGGACATTGA
q*SlLea*-R	TGTCTTCTTGTTTGTCACCGTTC
q*SlP5CS*-F	TGCTGTAGGTGTTGGTCGTCA
q*SlP5CS*-R	TGCCATCAAGCTCAGTTTGTG
q*Sl4CL*-F	GCTCTCTGCCCCGTAACCAA
q*Sl4CL*-R	CCACGATGAAAAGCTCATCATCAT
q*SlCHS1*-F	CAAAAGAAGGCCTAGGAACTA
q*SlCHS1*-R	CCCACTAAGCAGCAACACTG
q*SlDCL*-F	CCGCAAGGATGGTGAAACA
q*SlDCL*-R	TCCGCTTCCGAAAATGCC
q*SlGLK1*-F	GAATTTTCCGTAAGCAGTGGTG
q*SlGLK1*-R	CTTCTCCTTGATTTAGGCTCGT
q*SlSGR1*-F	ACTAGAAGGAAATGCAAGAAGAATCA
q*SlSGR1*-R	GCAACTTTCCTGGATGCTTTTC
q*SlHY5*-F	AGGTTAAAAAGATTGTTGAGAAATAGAG
q*SlHY5*-R	TGCACCTGCTGTTGTGTTCTT
q*SlPIF1*-F	GAACTAAGAAGTCCGCAATGGC
q*SlPIF1*-R	AGAGGGAATAACCGCATCACC
q*SlPIF3*-F	CCATCAGCCCCAAACTCACT
q*SlPIF3*-R	CTGCCATCTCATTATTGAACTCC
q*SlXTH5*-F	CCACCACCAGAGTGCGAGAT
q*SlXTH5*-R	TTTTCTTAGGATGACGATGTCCG
q*SlXTH7*-F	ATCCCCATTTTACGCATACTACA
q*SlXTH7*-R	GGGCTCAGTTGTTGATAAGAAGG
q*SlCPS*-F	AGGTCTTGTTTTGGCTCCCC
q*SlCPS*-R	CAAGTAGTGATGGATGTCTCTGCC
q*SlGA20ox2*-F	TAAGAAGGATAAGGTGGTGAGGC
q*Sl GA20ox2*-R	CCGTAGTTTTCTGTTGAAGCCA
q*SlGAST1*-F	CAACAACAGAGAAATAACCAAC
q*SlGAST1*-R	TTATACGATGTCTTTGAACACC
q*SlGID1*-F	ACCAAGGATGAATGGGTTTGTA
q*SlGID1*-R	TCTGTTCTGTTTTTACCGCTGA
q*SlCOI1*-F	CAACGATGGAAATAATGCAGAAAC
q*SlCOI1*-R	TGGACAAGACACCTAAAAAGGAAG
q*SlMYC2*-F	GGGTTCTGGTTCATGGGC
q*SlMYC2*-R	TTGCTGAGGAGGATTCTTCTGT
q*SlPYL2*-F	TCTCTTGTGACGCAACGCAT
q*SlPYL2*-R	GCTACCAACTCCGCCATCA
