# Supplementary material for: Generating drought- and salt-tolerant tomato germplasm through bioengineering of the ethylene response factor SlERF.J2
Source: Eng Life Sci. 2026 Jun 27;26(5-6):e003. doi: 10.48130/els-0026-0003 (PMC13599428; doi:10.48130/els-0026-0003)
Supplement: Supplementary file 1 [file els-2026-26-5-6-0003-S1.zip › 10.48130_els-0026-0003-Suppl-TableS2.pdf]

Supplementary Table S1. Details of Primers for PCR Amplification

Primer names	Primer sequence
CRSlERF.J2-F	ATTGCAGCATTAATGTGGAGTTG
CRSlERF.J2-R	AAACCAACTCCACATTAATGCT
NPTII-F	GACAATCGGCTGCTCTGA
NPTII-R	AACTCCAGCATGAGATCC
U6-26p-F	TGTCCCAGGATTAGAATGATTAGGC
U6-26p-R	CCCCAGAAATTGAACGCCGAAGAAC
J-SlERF.J2-F	CGTGGAGTAATCAGTGTTGTTCA
J-SlERF.J2-R	GTTGGGCTTTATGCGGGT
AD-SlERF.J2-F	GGAATTCATGAATAGATTCCGTCAAGATTCTC
AD-SlERF.J2-R	CGGGATCCCTAAGCCGAACATATCCATTTGA
AbAi-SlCPS-F	GGGGTACCACTATCCAGTTGCCATCAGACC
AbAi-SlCPS-R	CCCAAGCTTTGTTCATCCGATTGGTGCTTAT
