# Supplementary material for: Generating drought- and salt-tolerant tomato germplasm through bioengineering of the ethylene response factor SlERF.J2
Source: Eng Life Sci. 2026 Jun 27;26(5-6):e003. doi: 10.48130/els-0026-0003 (PMC13599428; doi:10.48130/els-0026-0003)
Supplement: Supplementary file 1 [file els-2026-26-5-6-0003-S1.zip › 10.48130_els-0026-0003-Suppl-FigureS1.pdf]

**A**

TATATGATATGACAATACAATGAAAACATTAAACTTAACGTAATAAAGGAACTATCCAGTTGCCATCAGACCATTCTTGATGAACCCTC
AACTTATCAATAGAATAGGCAACGGGAAATAGACATAGTACTCTTGTTCAACTTTGGCAGTGATTACCCCAAACCACCATCCCTCATT
GGCATACACATCGTAGATTTCCATTGGCCTTCTTTCATGTGGAATAACAGGTGGACCTCTGAAACATGTACCGACTCCTCTAGCGGC
GCCGTCTTATCATCATTCACCAGTGTTTTGTATTTAACTTTGTAATGATAAGCACCAATCGGATGAACAATACTTGCTTGGTAGTAAGA
ACCTATATAGCCTTCTTCTTGGCTTGCTACTTCAATGTCATCATTAATTTGAAATATTAACGTTGTTTCTTGTTTCTTATATGCTGTCTCTC

**B**

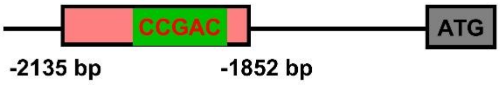


**Figure S1. Schematic diagram of cis-elements in the *SlCPS* promoter. A.** Sequence information of the cloned *SlCPS* promoter fragment. **B.** The cloned *SlCPS* promoter fragment is 283 bp in length and contains one DRE/CRT (CCGAC) cis-acting element.
